# Rupture of Uterus and Perforation of Uterus

**Published:** 1914-05

**Authors:** B. F. Kingsley

**Affiliations:** San Antonio, Texas


### TEXAS MEDICAL JOURNAL

Established July, 1885

F. E. DANIEL, M. D., - - - - Editor, Publisher and Proprietor

Published Monthly.—Subscription, $1.00 a Year.

VOL. XXIX. AUSTIN, MAY, 1914. No. 11.

The publisher is not responsible for the views of the contributors.

### Original Articles.

For Texas Medical Journal.

### "Rupture of Uterus and Perforation of Uterus."—Report of Two Cases.*

DR. B. F. KINGSLEY, SAN ANTONIO, TEXAS.

### RUPTURE OF THE WOMB.

*Case No. 1.*—This case was reported in part to this Society several years ago, but its final history and disposition are of sufficient interest to justify a more detailed report.

Was called one afternoon to see Mrs. — and obtained the following history: Age 23. Her first and only child was born five months perviously after a normal labor. During the time she nursed her child, had no menstrual period, but complained in a vague way of not feeling well since the birth of her child. Complained more than usual one day. She sent for her physician, who upon examination found a small cyst protruding from the cervix. Taking it far granted that she was pregnant and about to abort, she was prepared for an exploration or curettement, placed on the table and a nurse proceeded to administer chloroform. While partly under its influence and as the attending physician had passed the curette into the womb she began to vomit and suddenly became pale, almost pulseless, and a mass which was quickly recognized as the intestines presented at the vulva.

I was quickly summoned. A hurried glance revealed the situation, and she was sent to the hospital as quickly as possible. The

*Read before the Fifth District Association, November 6, 1913.

abdomen was opened and a mass of intestines filling the uterine cavity presented. The womb was as large as a womb pregnant four months. The rent extended entirely across the fundus, fully three inches long, admitting several coils of intestines which passed down through the womb, cervix and vagina. These were held firmly in the grasp of the contracted womb, the intestines had become of a port-wine color and it became necessary to incise one angle of the rent in order to release the gut, which was soon accomplished. There was only a small quantity of blood in the abdominal cavity which was thoroughly walled off in the beginning. There was no sign of placenta or membrane. The uterine cavity was well cleansed, and a gauze drain left in the cavity extending into the vagina. The wound was then closed with a line of deep chromic catgut sutures No. 2 interrupted, and also a second line of No. 0 interrupted through the peritoneum, and after looking after the pelvic and abdominal toilet the abdomen was closed as usual. The patient made a prompt and uninterrupted recovery, and I understand has since borne another child.

The case is interesting for several reasons. First the nature and extent of the rupture would seem to preclude the perforation theory. The size of the womb, the cervical cyst, would seem to convey the impression that there must have been a pregnancy and an abortion, but this was denied, the woman not being even suspicious of pregnancy, yet there was nothing else to account for the enlarged uterus. As we have seen, it must have been pretty large to admit several coils of intestines, and to enable them to pass through the cervix and vagina, yet there was no pelvic, uterine or abdominal evidence of pregnancy. Then what was the cause of this rupture? The most probable explanation to my mind is that the muscular rigidity incident to the early stage of the anesthetic, and the powerful intra-abdominal pressure induced by vomiting, were the chief factors.

This case emphasizes the necessity of being extremely careful in the use of the curette, as the uterine walls become very much attenuated from various growths and pathological processes which enlarge the uterus. It also indicates in the most forcible manner the line of treatment imperatively demanded in such cases; namely, abdominal section with the least possible delay. No other treatment is for a moment to be thought of. A delay of four or five hours with the intestines held in a grip sufficient to cut off the circulation, would probably have necessitated a resection of the bowels and destroyed the last hope of saving her life.

PERFORATION OF THE WOMB.

*Case No. 2.*—Mrs. —. A multipara, aged 27, missed two periods. Soon after which she began to flow and continued for several weeks. She finally called a physician who thought it advisable and necessary to curette. In doing so he passed the curette into the womb its full length, much to his surprise. He hereupon desisted from further efforts and awaited further developments. In the course of a few hours she had a severe chill, high fever, and a little later still followed by distention and soreness over the lower portion of the abdomen. These symptoms gradually increased, until at the expiration of about twelve hours I saw the pateient and sent her to the hospital. Abdominal section was done as quickly as possible and a perforation the size of an ordinary curette discovered in the right upper anterior surface of the fundus, which was bleeding slowly. The pelvic cavity was filled with blood and fluid and the pelvic organs and tissues were already matted together, and plastic inflammation was spreading rapidly upward over the intestines, omentum and peritoneum.

The perforation was closed by two deep and three peritoneal chromic catgut sutures. The pelvic and peritoneal cavities were then carefully cleansed of all clots and fluid by means of hot saline sponges, and the abdomen closed in the usual manner.

An iodoform pack was then placed in the uterus and also in the vagina, and the patient sent home in an ambulance.

In about thirty-six hours, accompanied by her family physician, I removed the packs, with a three months old macerated fetus. The womb was washed out with normal saline solution and she was given an ampoule of pituitrin to contract the thin and flabby uterus. Her recovery was prompt and uneventful.

The case is interesting for several reasons. First, the thin and flabbly uterine wall which rendered very little obstacle to the passage of a probe or curette, thus escaping the tactile sense of the physician, and in spite of his suspicion lulling him into sense of security until the constitutional symptoms forced upon him the reluctant conviction of perforation.

Second, the sequelae of events, whether developing slowly or rapidly after the use of the curette, should almost certainly lead the wide-awake physician to correct diagnosis, yet these symptoms may again be due to an acute infection following a curettement, but would usually develop much more slowly. Owing to the tardy recognition of the situation, the reluctance to have a misfortune demonstrated, or the absence of a skilled surgeon, the patient is usually permitted to die and the true condition never known, or until pelvic abscess forms with its accompanying or consequent adhesions and more or less permanent invalidism.

The lesson this case teaches and emphasizes is that in enlarged conditions of the womb, whether from pregnancy or pathological conditions, that organ often becomes very thin at some point and friable and easily punctured, hence the necessity of exercising the greatest possible care and delicacy in using the curette.

And third, the imperative necessity of prompt surgical interference by abdominal section as the only possible hope of the greater number of these cases.

In closing, let me say that I know of no instrument used with less scientific skill and precision, and the lack of which is fraught with more evil consequence, than the curette.

